# Alternatives to emergency departments for mental health crisis - a system wide approach can lead to better patient outcomes

**DOI:** 10.1192/j.eurpsy.2022.711

**Published:** 2022-09-01

**Authors:** J. Dove, S. Leveson

**Affiliations:** 1 Camden & Islington NHS Foundation Trust, Liaison Psychiatry, London, United Kingdom; 2 Camden & Islington NHS Foundation Trust, Liaison Psychiatry And Health Based Place Of Safety, London, United Kingdom

**Keywords:** covid, liaison, emergency, police

## Abstract

**Introduction:**

In Camden and Islington (North Central London) we have restructured our emergency mental health services significantly. Prior to January 2020 all emergency mental health presentations, including those detained in public by the police (S136) were supported through our three emergency departments and their respective liaison mental health teams. In January 2020 a new ‘Health Based Place of Safety’ (for those detained by police) was opened to avoid people spending time in emergency departments unnecessarily. When the COVID-19 pandemic first took hold in the UK in March 2020 a second unit, a ‘Mental Health Crisis Assessment Service’ (MHCAS) was set up again away from the acute sites, encouraging people in MH crisis to attend a designated MH ED away from the acute sites. This study aims to review the system and patient outcomes since the development of the pathway.

**Objectives:**

Relieving pressures on ED by reduction in patient numbers that could be better supported elsewhere and free up resource for alternative assessments and patient needs.

**Methods:**

A retrospective cohort study to review the outcomes of the new system in relation to emergency mental health crisis presentations. Comparison to be made with ED data for 2 years prior to new system.

**Results:**

Pending final results but initial data suggests 25% reduction in ED presentation for MH cause with new system. Reduction in psychiatric inpatient admissions of between 3-5%.

**Conclusions:**

Creative system wide initiatives to provide alternatives to emergency departments for people in emergency mental health crisis can lead to significantly improved patient outcomes and experience.

**Disclosure:**

No significant relationships.

